# Physiology and Pathophysiology of the Placenta

**DOI:** 10.3390/ijms24109066

**Published:** 2023-05-22

**Authors:** Giovanni Tossetta

**Affiliations:** Department of Experimental and Clinical Medicine, Università Politecnica delle Marche, 60126 Ancona, Italy; g.tossetta@univpm.it

We are pleased to present this Special Issue of the *International Journal of Molecular Sciences*, entitled “Physiology and Pathophysiology of Placenta”.

Placentation is an important and tightly regulated process that ensures the development of placenta, allowing for the normal progression of the fetus. The placenta is an essential organ which plays different and fundamental functions during pregnancy, and its development is regulated by several growth factors, hormones, their receptors and many other types of molecules involved in the regulation of placental cell proliferation, differentiation, migration and invasion [1,2].

The correct function of these processes is tightly regulated by the activation or inhibition of several signalling pathways that regulate the expression of specific genes necessary for a successful pregnancy. The importance of normal placental development becomes evident in the case of impaired placental development, which can lead to significant pregnancy complications such as preeclampsia (PE) [3], fetal growth restriction (FGR) [4], gestational trophoblastic diseases (GTD) [5], preterm delivery [6,7] and gestational diabetes mellitus (GDM) [8]. Pregnancy can also be impaired by exposure to exogenous agents such as bacteria [7], viruses [9], chemicals and natural compounds [9,10] that can alter the normal placental functions, compromising pregnancy outcome. Many of the disorders/pathologies previously mentioned are associated with an increase in maternal and fetal mortality and morbidity, and can lead to life-long health complications for both mother and child.

Important signalling pathways such as Wnt/-catenin, TGF/SMAD, PI3K/AKT/mTOR and JAK/STAT pathways have been reported to be impaired in several pregnancy complications such as PE, GDM and FGR [10,11,12,13], which share an inflammatory and oxidative stress condition [6,8]. In addition to the previously mentioned pathologies, viral and bacterial infections during pregnancy can also lead to an increase in inflammatory cytokines that alter the normal function of the placenta and amniotic membranes, causing preterm delivery or significant neonatal complications [7,9,14]. All the pathologies already mentioned cause systemic inflammation (acute or chronic) that turns into endothelial dysfunction. Endothelial dysfunction impairs the normal functionality of the endothelium, and thus can alter the normal function of reproductive organs [15,16,17,18].

For these reasons, new and specific biomarkers are necessary in clinical practice to allow an early diagnosis of many of the above-mentioned pregnancy complications, in order to carry out early treatment of the pathology, improving the outcome of the pregnancy or resolving the pathology [8,19,20].

Several natural and synthetic compounds have shown important beneficial effects in treating several diseases. These compounds have also demonstrated important effects in pregnancy complications, suggesting a possible use of these compounds, alone or in combination with classical drugs, to treat these diseases, improving pregnancy outcomes [21,22].

Understanding the mechanisms involved in the regulation of human placenta development in normal and pathological conditions can help to open new perspectives in the treatment of these pregnancy complications.

Thus, the aim of this Special Issue is to provide an overview of the physiology and pathophysiology of the placenta, in order to better understand its development in normal and pathological conditions.

## References

[1] Tossetta G., Avellini C., Licini C., Giannubilo S.R., Castellucci M., Marzioni D. (2016). High temperature requirement A1 and fibronectin: Two possible players in placental tissue remodelling. Eur. J. Histochem..

[2] Marzioni D., Crescimanno C., Zaccheo D., Coppari R., Underhill C.B., Castellucci M. (2001). Hyaluronate and CD44 expression patterns in the human placenta throughout pregnancy. Eur. J. Histochem..

[3] Tossetta G., Fantone S., Giannubilo S.R., Marinelli Busilacchi E., Ciavattini A., Castellucci M., Di Simone N., Mattioli-Belmonte M., Marzioni D. (2019). Pre-eclampsia onset and SPARC: A possible involvement in placenta development. J. Cell Physiol..

[4] Cardaropoli S., Paulesu L., Romagnoli R., Ietta F., Marzioni D., Castellucci M., Rolfo A., Vasario E., Piccoli E., Todros T. (2012). Macrophage migration inhibitory factor in fetoplacental tissues from preeclamptic pregnancies with or without fetal growth restriction. Clin. Dev. Immunol..

[5] Marzioni D., Quaranta A., Lorenzi T., Morroni M., Crescimanno C., De Nictolis M., Toti P., Muzzonigro G., Baldi A., De Luca A. (2009). Expression pattern alterations of the serine protease HtrA1 in normal human placental tissues and in gestational trophoblastic diseases. Histol. Histopathol..

[6] Cecati M., Sartini D., Campagna R., Biagini A., Ciavattini A., Emanuelli M., Giannubilo S.R. (2017). Molecular analysis of endometrial inflammation in preterm birth. Cell Mol. Biol. (Noisy-Le-Grand).

[7] Licini C., Tossetta G., Avellini C., Ciarmela P., Lorenzi T., Toti P., Gesuita R., Voltolini C., Petraglia F., Castellucci M. (2016). Analysis of cell-cell junctions in human amnion and chorionic plate affected by chorioamnionitis. Histol. Histopathol..

[8] Tossetta G., Fantone S., Gesuita R., Di Renzo G.C., Meyyazhagan A., Tersigni C., Scambia G., Di Simone N., Marzioni D. (2022). HtrA1 in Gestational Diabetes Mellitus: A Possible Biomarker?. Diagnostics.

[9] Tossetta G., Fantone S., Delli Muti N., Balercia G., Ciavattini A., Giannubilo S.R., Marzioni D. (2022). Preeclampsia and severe acute respiratory syndrome coronavirus 2 infection: A systematic review. J. Hypertens..

[10] Alijotas-Reig J., Esteve-Valverde E., Ferrer-Oliveras R., Llurba E., Gris J.M. (2017). Tumor Necrosis Factor-Alpha and Pregnancy: Focus on Biologics. An Updated and Comprehensive Review. Clin. Rev. Allergy Immunol..

[11] Villalobos-Labra R., Silva L., Subiabre M., Araos J., Salsoso R., Fuenzalida B., Saez T., Toledo F., Gonzalez M., Quezada C. (2017). Akt/mTOR Role in Human Foetoplacental Vascular Insulin Resistance in Diseases of Pregnancy. J. Diabetes Res..

[12] Zhang Z., Wang X., Zhang L., Shi Y., Wang J., Yan H. (2017). Wnt/beta-catenin signaling pathway in trophoblasts and abnormal activation in preeclampsia (Review). Mol. Med. Rep..

[13] Li Y., Yan J., Chang H.M., Chen Z.J., Leung P.C.K. (2021). Roles of TGF-beta Superfamily Proteins in Extravillous Trophoblast Invasion. Trends Endocrinol. Metab..

[14] Ikumi N.M., Matjila M. (2022). Preterm Birth in Women With HIV: The Role of the Placenta. Front. Glob Womens Health.

[15] Mateuszuk L., Campagna R., Kutryb-Zajac B., Kus K., Slominska E.M., Smolenski R.T., Chlopicki S. (2020). Reversal of endothelial dysfunction by nicotinamide mononucleotide via extracellular conversion to nicotinamide riboside. Biochem. Pharmacol..

[16] Szczesny-Malysiak E., Stojak M., Campagna R., Grosicki M., Jamrozik M., Kaczara P., Chlopicki S. (2020). Bardoxolone Methyl Displays Detrimental Effects on Endothelial Bioenergetics, Suppresses Endothelial ET-1 Release, and Increases Endothelial Permeability in Human Microvascular Endothelium. Oxid. Med. Cell Longev..

[17] Campagna R., Mateuszuk L., Wojnar-Lason K., Kaczara P., Tworzydlo A., Kij A., Bujok R., Mlynarski J., Wang Y., Sartini D. (2021). Nicotinamide N-methyltransferase in endothelium protects against oxidant stress-induced endothelial injury. Biochim. Biophys. Acta Mol. Cell Res..

[18] Zapotoczny B., Braet F., Kus E., Ginda-Makela K., Klejevskaja B., Campagna R., Chlopicki S., Szymonski M. (2019). Actin-spectrin scaffold supports open fenestrae in liver sinusoidal endothelial cells. Traffic.

[19] Gesuita R., Licini C., Picchiassi E., Tarquini F., Coata G., Fantone S., Tossetta G., Ciavattini A., Castellucci M., Di Renzo G.C. (2019). Association between first trimester plasma htra1 level and subsequent preeclampsia: A possible early marker?. Pregnancy Hypertens..

[20] Licini C., Avellini C., Picchiassi E., Mensa E., Fantone S., Ramini D., Tersigni C., Tossetta G., Castellucci C., Tarquini F. (2021). Pre-eclampsia predictive ability of maternal miR-125b: A clinical and experimental study. Transl. Res..

[21] Tossetta G., Fantone S., Giannubilo S.R., Marzioni D. (2021). The Multifaced Actions of Curcumin in Pregnancy Outcome. Antioxidants.

[22] Novakovic R., Rajkovic J., Gostimirovic M., Gojkovic-Bukarica L., Radunovic N. (2022). Resveratrol and Reproductive Health. Life.

